# Serovar D and E of serogroup B induce highest serological responses in urogenital *Chlamydia trachomatis* infections

**DOI:** 10.1186/1471-2334-14-3

**Published:** 2014-01-02

**Authors:** Stephan P Verweij, Esmée Lanjouw, Caroline J Bax, Koen D Quint, Paul M Oostvogel, P Joep Dörr, Jolein Pleijster, Henry JC de Vries, Remco PH Peters, Sander Ouburg, Servaas A Morré

**Affiliations:** 1 Laboratory of Immunogenetics, Department of Medical Microbiology and Infection Control, VU University Medical Center, De Boelelaan 1117, 1081, HV, Amsterdam, The Netherlands; 2 Department of Dermatology, Erasmus Medical Center Rotterdam, Rotterdam, The Netherlands; 3 Department of Obstetrics and Gynecology, MC Haaglanden Hospital, The Hague, The Netherlands; 4 Department of Obstetrics, VU University Medical Center, Amsterdam, The Netherlands; 5 DDL Diagnostic Laboratory, Rijswijk, The Netherlands; 6 Department of Dermatology, Leiden University Medical Centre, Leiden, The Netherlands; 7 Department of Medical Microbiology, MC Haaglanden Hospital, The Hague, The Netherlands; 8 STI outpatient clinic, Cluster Infectious Diseases, Public Health Service Amsterdam, Amsterdam, The Netherlands; 9 Department of Dermatology, Academic Medical Center, University of Amsterdam, Amsterdam, The Netherlands; 10 Centre for Infection and Immunity Amsterdam (CINIMA), Academic Medical Center, University of Amsterdam, Amsterdam, The Netherlands; 11 The Hague Municipal Health Service, The Hague, The Netherlands; 12 Anova Health Institute, Khutšo Kurhula Offices, Tzaneen, South Africa; 13 Institute of Public Health Genomics, Department of Genetics and Cell Biology, Research Institute GROW, Faculty of Health, Medicine & Life Sciences, University of Maastricht, Maastricht, The Netherlands

**Keywords:** *Chlamydia trachomatis*, Serological response, Serovar, Serogroup, IgG, Antibodies

## Abstract

**Background:**

*Chlamydia trachomatis* is the most prevalent bacterial sexually transmitted infection (STI) worldwide. A strong link between *C. trachomatis* serogroup/serovar and serological response has been suggested in a previous preliminary study. The aim of the current study was to confirm and strengthen those findings about serological IgG responses in relation to *C. trachomatis* serogroups and serovars.

**Methods:**

The study population (n = 718) consisted of two patient groups with similar characteristics of Dutch STI clinic visitors. We performed genotyping of serovars and used titre based and quantitative commercially available ELISA kits (medac Diagnostika) to determine specific serum IgG levels. Optical density (OD) values generated by both tests were used to calculate the IgG titres (cut-off 1:50). Analyses were conducted stratified by gender.

**Results:**

We observed very significant differences when comparing the median IgG titres of three serogroups, B, C and I: in women for B *vs.* C: *p* < 0.0001 (median titres B 200 *vs.* C <50); B *vs.* I: *p* < 0.0001 (200 *vs.* 50), and in men for B *vs.* C: *p* = 0.0006 (150 *vs.* <50); B *vs*. I: *p* = 0.0001 (150 *vs.* <50); C *vs.* I was not significant for both sexes. Serovars D and E of serogroup B had the highest median IgG titres compared to the other serovars in both men and women: 200 and 200 *vs*. ≤ 100 for women and 100 and 200 *vs*. ≤ 75 for men, respectively.

**Conclusions:**

This study shows that B group serovars induce higher serological responses compared to the C and I group serovars *in vivo* in both men and women.

## Background

*Chlamydia trachomatis* is the most prevalent bacterial sexually transmitted infection (STI) worldwide. The clinical course of infection is frequently asymptomatic resulting in patients not seeking treatment. Untreated urogenital *C. trachomatis* infections may give rise to late complications, including pelvic inflammatory disease, ectopic pregnancy, and sub- or infertility [1]. Assessment of serological responses to the bacterium resulted in a classification in serovars [2]. Nowadays, 19 different serovars are known, usually causing conjunctival and urogenital infections. The serovars are subdivided in three serogroups based on phylogenetic comparisons of the *ompA* gene. Serogroup B contains serovars B, Ba, D, Da, E, L1, L2, L2a; serogroup C contains: A, C, H, I, Ia, J, K, L3; serogroup I contains: F, G, Ga [3,4]. Identification and typing of serovars is currently done by rapid PCR-based techniques [5-7]. Geographical distribution of the serogroups is very similar worldwide on a national level, but there are variations in distribution within cities [8,9]. The most prevalent serovars worldwide are D, E, and F [10,11].

Several studies have shown associations between urogenital serovars and severity of clinical symptoms and the course of *C. trachomatis* infections [11,12], although conflicting data have also been reported [13,14]. It has been shown that serovar K is associated with abnormal vaginal discharge, and serovar Ga is associated with dysuria in men [12]. Van Duynhoven *et al.*[11] reported that infections with serovars D/D-, H, and K appeared to be associated with inflammation, as shown by the presence of 10 or more leukocytes per microscopic field (magnification, x400). This was determined in first-void urine samples from men and in gram-stained cervical smears from women. However, this was not statistically significant. A comparison study of serovars D and H in a murine model shows that serovar H elementary bodies (EB) are more cytotoxic than EBs of serovar D, but the serovar D EBs have a longer duration of infection [15]. In all studies however, the B group serovars are most prevalent and the C group serovars least common, suggesting different immunological responses by the host to serovars/serogroups. This may be of clinical interest since an association was observed between higher antibody responses and severity of tubal pathology [16].

In a previous study [17] we measured immunoglobulin (Ig) G titres in 235 *C. trachomatis* infected patients. For these measurements we used a quantitative ELISA kit developed by medac Diagnostika. We observed that the most prevalent serogroup B induced significantly higher serological IgG responses than serogroups C and I [17]. These results suggest a relation between serovars and the serological responses in human and potentially offer a partial explanation of the geographical prevalence of specific serovars. These results, however, have not been verified in different patient populations, nor have they been confirmed using other serological tests.

The aim of this study was to confirm and expand our previous study concerning serological IgG responses in relation to *C. trachomatis* serogroups and serovars. We used a study population consisting of two different patient groups and for our experiments we made use of titre-based and quantitative serological ELISA assays. We explored whether or not the most prevalent serovars (D, E, and F) induced the highest serological responses, in addition to analysing at serogroup level only as has been done previously [17].

## Methods

### Patient populations and *C. trachomatis* detection

The study included samples from 718 *C. trachomatis* infected patients, derived from two different patient groups. The first group (Study 1) was obtained from 357 microbiologically confirmed *C. trachomatis* patients (as determined by PACE2 assay, Gen Probe) obtained from the STI outpatient clinic (n = 312) and the department of obstetrics and gynaecology (n = 45) in The Hague, the Netherlands. The samples include cervical and/or vaginal swabs, urethral swabs, and urine specimens. Serum samples were collected from all patients. These samples were stored in the period January to October 2008. Reasons for patients visiting either the STI clinic or gynaecology department and the collection of demographic data are described elsewhere [17]. To prevent possible bias in serological responses we excluded patients with a multiple site infection or mixed infection, and patients with an infection other than the urethral tract or cervicovaginal tract (n = 70). A total number of 287 patients (149 women and 138 men) were used for analyses, of which 82 were unique compared to the previous study [17].

**Figure 1:**
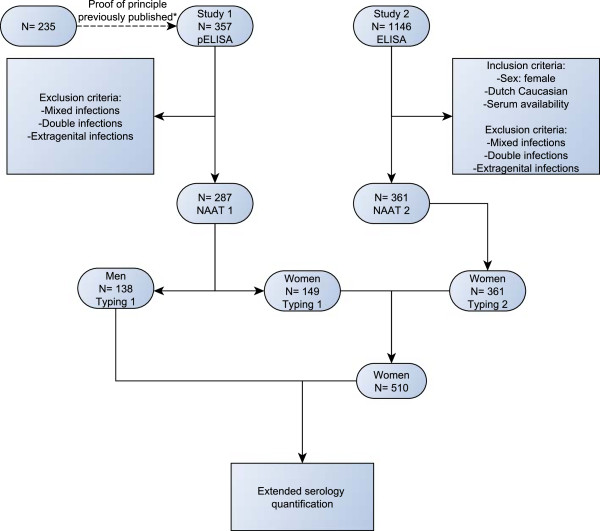
**The figure shows a flowchart summarising the in- and exclusion criteria and the groups analysed.** *: reference [17]. NAAT 1: PACE2 assay. NAAT 2: COBAS AMPLICOR. Typing 1: *C. trachomatis*-DT assay. Typing 2: sequencing VD2.

The second patient group (Study 2) included 361 *C. trachomatis* infected women (as determined by COBAS AMPLICOR, Hoffman – La Roche, Basel, Switzerland). Characteristics are described elsewhere [18]. In short, cervical swabs were taken and serum was collected from women attending an STI outpatient clinic in Amsterdam, the Netherlands. Demographical and clinical data were available and informed consent was obtained [18]. Ethnicity in both patient groups was self-reported, which has been shown a highly valid and representative marker of ethnicity in this context [19]. Figure 1 shows a flowchart summarising the in- and exclusion criteria and the groups analysed.

### Ethical approval

The Medical Research Involving Human Subjects Act (WMO, Dutch Law) stating official approval of the study by the Medical Ethical Committee does not apply to our anonymous human material collected (MEC Letter reference: # 10.17.0046).

### *C. trachomatis* genotyping

Serogroup and serovar determination for Study 1 was performed with the *C. trachomatis*-DT assay (Labo Biomedical Products BV, Rijswijk, the Netherlands) as described elsewhere [8]. Shortly, DNA was extracted from the swabs and urine samples to detect *C. trachomatis* for confirmation of the PACE2 assay. After confirmation, genotyping was performed using a reverse hybridization probe line blot with a probe for the cryptic plasmid and a probe for the three serogroups and all serovars.

*C. trachomatis* genotyping in Study 2 was performed by means of PCR based RFLP analyses as described previously or, for the second half by sequencing the VD2 region of the *ompA* gene [20].

### *C. trachomatis* serology

Study 1: determination of *C. trachomatis* IgG levels in serum of all patients was done by pELISA (*Chlamydia trachomatis*-IgG-ELISA plus kit (medac Diagnostika, Germany)) and was performed according to the manufacturer’s protocols. This is an assay generating quantitative results (Arbitrary Units/ml), based on OD values. These OD values can also be used to calculate the titres. For this study, we calculated the titres to make the results in accordance with the results of Study 2. Cut-off levels for negative, grey zone, and positive *C. trachomatis* IgG serology were used as described by the manufacturer (cut-off 1:50).

Study 2: determination of IgG levels was done previously by *Chlamydia trachomatis*-IgG-ELISA (medac Diagnostika, Germany) according to the manufacturer’s protocol. Titres ≥ 1:50 were considered positive.

Similar titre calculations for both Medac assays were performed as described by the manufacturer’s instructions.

### Statistical analyses

Since different antibody levels against *C. trachomatis* have been described in men and women [21], we analysed men and women separately, except when stated differently. Mann–Whitney U tests were used to compare median titres between the serogroups (B *vs*. C; B *vs*. I; C *vs*. I). Mann–Whitney U tests were used to compare median titres of serovars within serogrous. Analyses were performed using Prof. Avery’s ‘*utest’* program [22]. *P* values < 0.05 were considered statistically significant.

## Results

Characteristics and serovar distribution between the two patient groups (Study 1 and 2) were compared. We compared serological distribution of the serogroups and serovars, and we compared age between the two individual patient groups. We did not observe any significant differences in distribution of serological titres between both patient groups in total, or between ethnic subgroups (general distribution: 57.3% Dutch Caucasian; 8.0% Surinam; 8.5% Dutch Antilles; 3.8% North African; 2.4% western European (non Dutch); 2.0% eastern European; 18.0% other nationalities). We observed no differences in mean age and distribution of the serological titres per serovar and serogroup between both patient groups in total, or between the ethnic subgroups. Therefore we combined both groups and analysed them as one.

### Serogroup analyses

**Figure 2:**
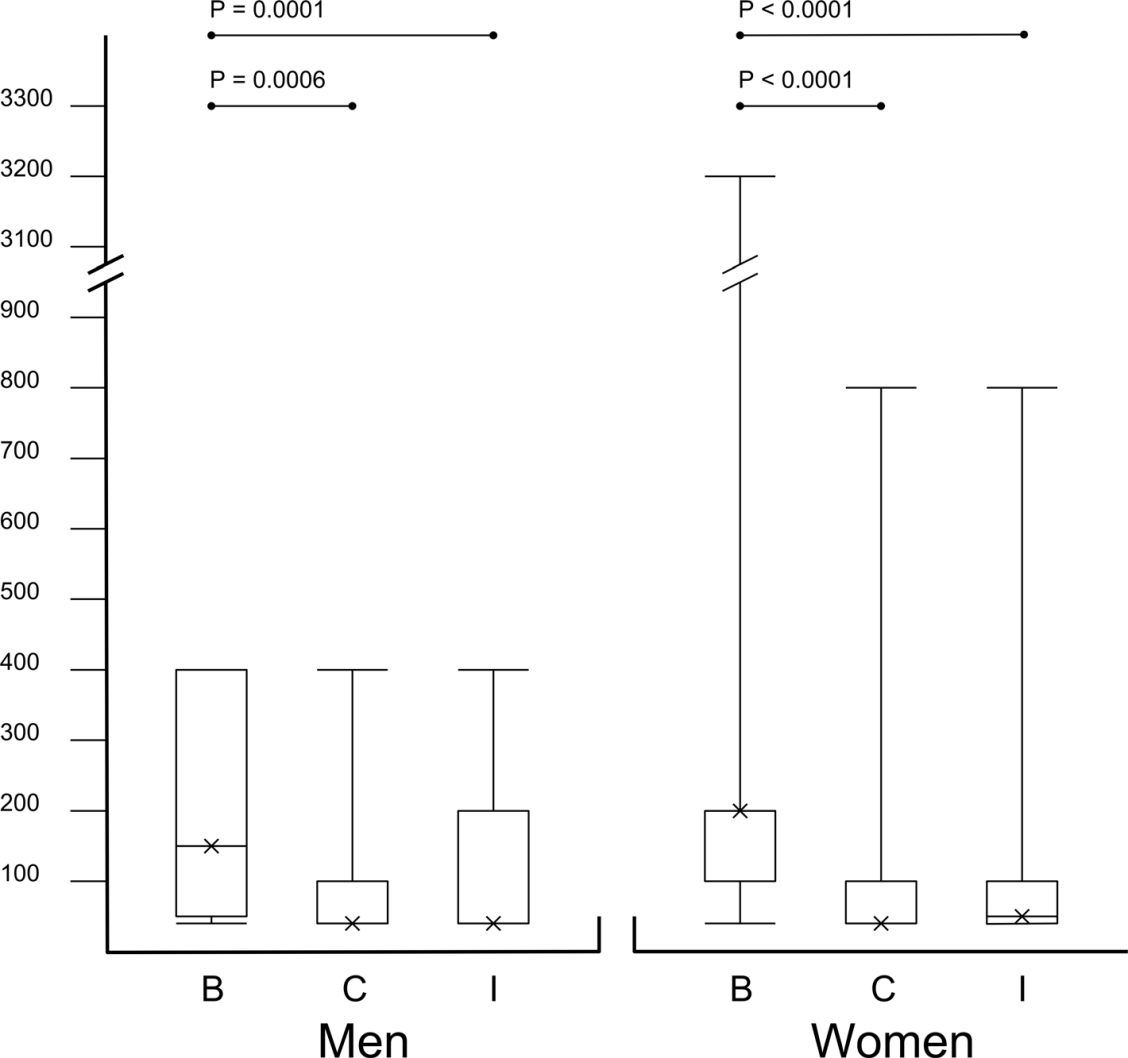
**The figure shows the median titres for three serogroups in men and women, depicted as a boxplot.** The upper and lower limit of the box represents the first and third quartile. The cross represents the median of the group. The upper and lower limit of the range is presented as horizontal bars on a vertical axis. The large range between upper and lower limits are mostly caused by single samples that might be considered outliers. The distribution of the titres is best represented by the medians, and the first and third quartile limits.

The serological titres per serogroup and per serovar are shown in Table 1 and Figure 2 for both men and women. Serogroup B had significantly higher median IgG titres compared to the C and I serogroups analysed in both groups. Female group: B *vs.* C: *p* < 0.0001 (*p*: 2.0×10^-6^; 200 *vs.* <50); B *vs*. I: *p* < 0.0001 (*p*: 2.0×10^-6^; 200 *vs.* 50); male group: B *vs.* C: *p* = 0.0006 (150 *vs.* <50); B *vs*. I: *p* = 0.0001 (150 *vs.* <50). No significant differences were observed between serogroups C and I. For women C *vs.* I: *p* = 0.14 (<50 *vs.* 50), for men C *vs.* I: *p* = 0.65 (<50 *vs.* <50).

**Table 1:** Median serological IgG titres in men and women subdivided by serogroup and serovar

**Serogroup**	**n**	**%**	**Median titre IgG**	**Range**	**Serovar**	**n**	**%**	**Median titre IgG**	**Range**
**Women (n = 510)**									
B	262	51.8	200*	<50 – 3200	D/Da/D-	45	8.8	200	<50 – 3200
					E	217	42.2	200	<50 – 1600
C	89	17.3	<50	<50 – 800	H	20	3.9	75	<50 – 800
					I/Ia/I-	36	7.0	<50	<50 – 400
					J	20	3.9	<50	<50 – 400
					K	13	2.5	100	<50 – 800
I	159	30.9	50	<50 – 800	F	127	24.7	50	<50 – 800
					G/Ga	32	6.2	100	<50 – 400
**Men (n = 138)**									
B	66	47.8	150^†^	<50 – 400	D/Da/D-	11	8.0	100	<50 – 400
					E	55	39.9	200	<50 – 400
C	23	16.7	<50	<50 – 400	H	0	0.0	n/a	n/a
					I/Ia/I-	3	2.2	50	<50 – 50
					J	12	8.7	<50	<50 – 200
					K	8	5.8	75	<50 – 400
I	49	35.5	<50	<50 – 400	F	30	21.7	50	50 – 400
					G/Ga	19	13.8	<50	<50 – 400

*: B-group significantly different from C and I group (*p* < 0.0001 and *p* < 0.0001) respectively. ^†^: B-group significantly different from C and I group (*p* = 0.0006 and *p* = 0.0001) respectively.

### Serovar analyses

Table 1 shows the median IgG titres per serovar for both women and men. At the serovar level in the female group, serovars D and E demonstrated the highest median IgG, followed by serovars K, H, G, and F. Serovars J and I showed the lowest titres. In the Dutch male group serovars D and E induced the highest median IgG titres, followed by serovar G, K, F, J, and I. Serovar H was not present in this group.

### In-group analyses

Within the serogroups, no difference was observed between serovars D and E in both men and women. For females, median IgG titres in serovar J infected patients were significantly lower compared to serovar K infected patients (*p* = 0.029; <50 *vs.* 100). No differences were observed between the other serovars of group C. In serogroup I (serovars F and G) no significant differences were observed in median IgG titres. No differences in serological responses were observed within the serogroups for the male group.

## Discussion

This study shows that the most prevalent urogenital *C. trachomatis* serogroup B causes the highest serological IgG responses. More specifically, both serovars D and E of the B serogroup show higher IgG serological responses than the other serovars. Within serogroup C of the combined Dutch female patient group, we found a small difference between serovar K and J. The results generated in this study may be of clinical interest since an association was observed between antibody response and severity of tubal pathology in an earlier study [16].

We observed higher serological responses in women compared to men for all serovars, but this did not reach statistical significance. Eighty-two samples from Study 1 were unique compared to the previous study after exclusion. These samples showed similar results as compared to the earlier samples [17]. Compared to our previous study [17] of 235 patients, we increased our total study group to 648 patients and differentiated between men and women and confirmed our preliminary results. Both of our studies were performed with the medac ELISA assays. Interestingly, we observed similar results in a group of *C. trachomatis* infected Danish women (n = 91) using a different *C. trachomatis*-specific serological IgG test (SeroCT IgG ELISA; Savyon Diagnostics Ltd., Ashdod, Israel) [23]; serogroup B serovars induced the highest serological response compared to the other serovars (unpublished data).

In general, serological *C. trachomatis* IgG assays perform equally in sensitivity and specificity for these STI cohorts as we have previously shown [23]. Recently a potentially new target for *C. trachomatis* serology has been evaluated, but this assay is not yet commercially available [24]. This pgp3 assay showed highest relevance for titre positivity over time as compared to other commercially available tests. In our study population no follow-up samples were obtained. It would be of interest to compare the pgp3 assay with the used medac assay since about 30% of the samples had *C. trachomatis* DNA present without a detectable serological response.

Current detection methods for *C. trachomatis* are based on PCR techniques for DNA or RNA amplification. We used two different methods for *C. trachomatis* detection in this study and combined the results. However, the sensitivities and specificities of the current detection methods are very comparable, so no potential bias was introduced [25]. We also used two different serovar determination techniques. Quint *et al.* compared the *C. trachomatis*-DT assay to sequencing of the VD2 region for *C. trachomatis* genotyping. They found equal performances for both methods, so no potential bias was introduced in this study by making use of two different genotyping methods [26].

To gain more knowledge about differences in serological IgG responses between serovars in humans, studies in murine models and at cellular level may give more insight in this biological mechanism. Ito *et al.*[27] suggested in an earlier study that high serological responses in mice induced by the B serogroup (D and E) may be caused by longer antigenic stimulation, implicating that these high responses just reflect duration of infection. Lyons *et al.*[15] compared the cytotoxicity between the serovars D and H. They found that serovar H is much more cytotoxic than serovar D. Results of these studies suggested that serovars D and E (of the B serogroup) induce higher serological IgG responses in mice and have a longer duration than the C and I serogroup serovars. In contrast, serovar H is more cytotoxic than serovar D, but induces a lower IgG titre in the murine model. Eckert *et al.*[28] studied median IFU counts, a measure for virulence, in McCoy cells inoculated with *C. trachomatis* infected human urethral and cervical specimens. They observed that serovars B, D, and E induced the highest amount of IFU, and serovars I and J induced the smallest amount of IFU. This is a similar trend to what we find with regard to the serological responses in our study population.

By using a large group of patients, this study confirmed our previous results regarding serological responses and based on the size of the study population we were also able to link back to the serovar level [17]. Our results bring us one step closer to understanding the immunological response against a *C. trachomatis* infection at both serogroup and serovar level, and the variation in serological responses between women and men. From a clinical point of view it would be interesting to identify a relation between serological responses and *C. trachomatis* serovars/serogroups. However, none of the limited human studies performed on *C. trachomatis* serovars and the clinical course of infection included serological responses, making it currently impossible to translate our finding into a direct application for the clinic [29-31]. The knowledge generated in this study may aid in the development of a vaccine against *C. trachomatis* infection for which immunological responses to specific serovars linked to the natural duration of infection might be useful.

## Conclusions

In conclusion, this study shows that serogroup B induces the highest serological responses compared to the other serogroups. More specifically, serovars D and E show higher serological responses than the other serovars.

## Competing interests

The authors declare that they have no competing interests.

## Authors’ contributions

SPV: Sample analysis, data/statistical analysis, drafting the manuscript. EL: data/statistical analysis, drafting the manuscript. CJB: Sample collection, data collection, analysing data, critically reading manuscript. KDQ: Sample analysis, critically reading manuscript. PMO: Sample collection, critically reading the manuscript. PJD: Sample collection, critically reading the manuscript. JP: Sample analysis, critically reading manuscript. HJCV: Sample collection, critically reading the manuscript. RPHP: data collection, critically revising the manuscript. SO: data/statistical analysis, coordinating SPV and EL, critically revising the manuscript. SAM: study design, conception and coordination, critically revising the manuscript. All authors contributed to writing of the final manuscript. All authors read and approved the final manuscript.

## Pre-publication history

The pre-publication history for this paper can be accessed here:

http://www.biomedcentral.com/1471-2334/14/3/prepub
